# Congenital Nasal Bones Agenesis: Report of a Rare Malformation

**DOI:** 10.1155/carm/1849957

**Published:** 2024-12-23

**Authors:** Monica Russo, Chiara Ferrecchi, Silvia Rebella, Valeria Capra, Franco Ameli, Mattia Pacetti, Maria Francesca Di Feo, Pierangela De Biasio, Cesare Arioni

**Affiliations:** ^1^Operative Unit of Neonatology, IRCCS Ospedale Policlinico San Martino, Genoa, Italy; ^2^Department of Neurosciences, Rehabilitation, Ophthalmology, Genetics and Maternal and Child Sciences (DINOGMI), University of Genoa, Genoa, Italy; ^3^Genomics and Clinical Genetics, IRCCS Istituto Giannina Gaslini, Genoa, Italy; ^4^Otorhinolaringology Department, Casa di Cura Villa Montallegro, Genoa, Italy; ^5^Department of Neurosurgery, IRCCS Istituto Giannina Gaslini, Genoa, Italy; ^6^Head Fetal Medicine Unit, IRCCS Ospedale Policlinico San Martino, Genoa, Italy

**Keywords:** agenesis, arhinia, genetic, hyporhinia, malformation, nasal bones, neonatology

## Abstract

Congenital arhinia and hyporhinia are rare facial anomalies whose knowledge usually comes from case reports. The severity of each case described in literature is variable; it also depends on associated malformations too. Since the newborns are obligate nasal breathers, babies with arhinia or hyporhinia usually have respiratory distress and need airway stabilization. In addition, most of these children present difficulties in feeding and this impairment must be managed early. We describe an unusual case of partial congenital arhinia, the baby did not have other anomalies or any specific complication such as respiratory and feeding issues, so the major problem was the aesthetic and psychological issues for the family. Even if the neonatal course was uncomplicated, a coordinated approach of the pediatrician with the pediatric otolaryngologist, the geneticists and the neurosurgeons was necessary because the management of these malformations is always very complex; due to the lack of reports described in literature, an univocal management and also the best timing and technique for reconstructive surgery are still not defined.

## 1. Introduction

Congenital anomalies of the nose are relatively rare craniofacial malformations, affecting approximately 1 child every 20.000–40.000 live births [1]. The spectrum of congenital nasal malformations is varied [2] and they can result in abnormalities of function, appearance, or both.

Congenital arhinia and hyporhinia (complete or partial absence of the nose) are extremely rare. The pathophysiology of these malformations is still poorly understood, but several possible causes have been reviewed in the literature [3, 4]. Genetic causes are often unknown, but the literature presents some genetic or familial cases involved in these malformations [5, 6].

Phenotypic expression ranges from hyporhinia, manifested by the lack of external nasal structures, to total arhinia, characterized by a failure of formation of the external nose, nasal airways, olfactory bulbs, and olfactory nerve [7, 8].

Severe arhinia can cause a significant higher rate of perinatal mortality and morbidity due to severe upper airway obstruction, increased risk of respiratory distress, infections, and poor feeding. Furthermore, arhinia and hyporhinia represent a significant stress for families and this malformation will impact on children themselves when they will be adolescents, because of the aesthetic and psychological issues that negatively influence quality of life and mental health. The majority of the knowledge on these malformations comes from observation of patients and case reports. In consequence, there is not a global consensus regarding the correct management, leaving many doubts about classification, pathogenesis, and the best timing for reconstructive surgery. A patient with partial congenital arhinia is presented, and the diagnosis and management are discussed.

## 2. Case Presentation

**Figure 1:**
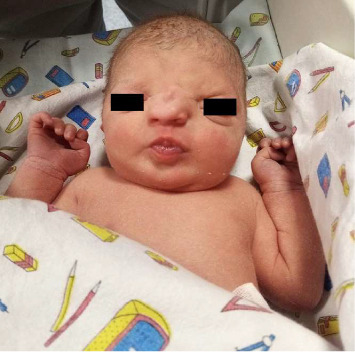
Newborn facial dysmorphia.

**Figure 2:**
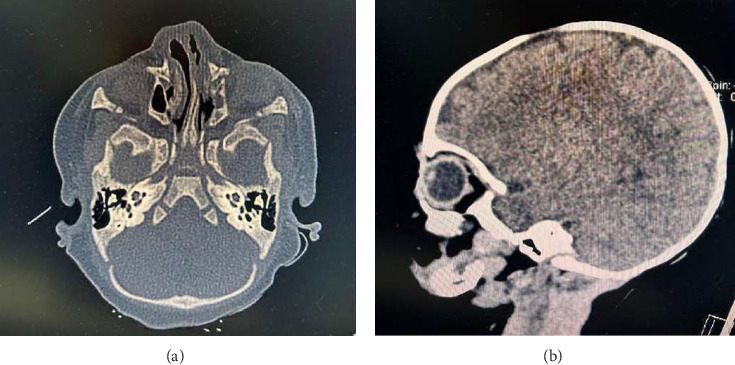
Coronal (a) and sagittal (b) computed tomography (CT) scans of the craniofacial skeleton.

**Figure 3:**
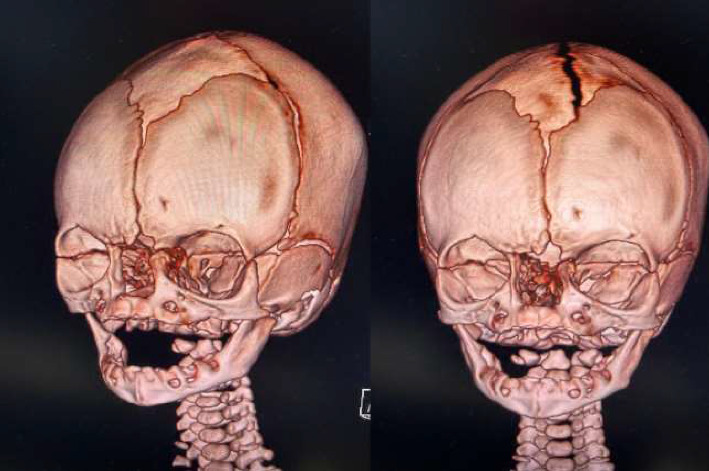
Facial three-dimensional computed tomography (3D-CT) scans.

A live Caucasian female infant was born to a 30-years-old mother with no significant medical history (gravida 0, para 0, abortus 0), the pregnancy was spontaneous. She was born by spontaneous vaginal delivery at term (39 + 2 weeks of gestation), with adequate weight (3295 gr, 60°PC), length (50 cm, 66°PC), and head circumference (35.5 cm, 93°PC) for gestational age according to INeS Charts. The prenatal course, followed in a different obstetric center, was physiological accomplished without complications. Fetal ultrasounds and combined test were normal. No history of consanguinity or a family history of congenital craniofacial malformations. The mother was not exposed to drugs, alcohol or teratogens during the pregnancy. Maternal history was also negative for diabetes mellitus, hypertension, and infections. At birth, the patient was in good condition and had immediately normal breathing through her mouth, so she only required routine assistance. The Apgar scores were 9 and 10 at 1 and 5 min, respectively. On first clinical examination the baby showed facial abnormality with depressed nasal bridge with abundant overlying skin and narrow and asymmetrical nostrils. The middle vault seemed collapsed and the nasal ala and tip were deformed. The nasal philtrum was broad and flat. Low ear implant and palpebral asymmetry with hypertelorism were associated too (Figure 1). Nasal examination was completed by palpation: the bones did not seem aligned, but spaced apart. At the complete physical evaluation no other associated malformations were noticed, neurological evaluation was normal too. Nasal patency was confirmed inserting a tube into the choanae. The baby was temporarily admitted in the Neonatal Pathology Unit to monitor vital signs and assess breastfeeding: impaired breathing or feeding difficulties were not present and the baby could adapt oral breathing and suck and breath simultaneously. So, after 24 h, the newborn was moved in rooming-in with her mother meanwhile further investigations were carried out and parents were referred for a psychological counseling. Red reflex and acoustic otoemissions, as well as cranial ultrasound, ophthalmology evaluation and *auditory brainstem response* (ABR) monitoring were normal. Septic screen and other blood investigations (complete blood count, C-reactive protein, renal and liver function test, and serum electrolytes) were normal too. Pediatric otolaryngology consult was performed, nasal and superior airway fibroscopy showed widened maxillar frontal processes and lack of nasal bones fusion, asymmetry of the alar cartilages, incomplete growth of the triangular cartilages, severe septal deviation on the left side without complete obstruction of the anterior nasal respiratory space, nasal lateral wall, and nasopharynx. The evaluation of blood vessel pattern and of quality of nasal mucosa was normal, just like the colour and the properties of the secretions. The child was referred for neurosurgical evaluation too. A brain and maxillofacial computed tomography (CT) scan was performed, it did not reveal sphenoid meningocele or basiocranio lesions, but nasal bones agenesis, frontal (glabella) dysplasia and verticalization of maxillar nasal processes (Figure 2). To perform a correct diagnosis and treatment plan, the patient was subjected to a 3D-CT reconstruction too, which improved the evaluation of the face and nasal bones (Figure 3). Genetic counseling was not conclusive, karyotype and Array-CGH were normal, while Whole-Exome Sequencing (WES) did not detect any pathogenic genetic variant. Neurosurgical evaluation concluded that immediate surgical approach was not needed and the baby was referred to the follow-up.

## 3. Discussion

### 3.1. Definition

Arhinia and hyporhinia refer to congenital failure of the normal nasal development. In his review, Cohen et al. provided a clear classification system for this malformation suggesting to designate the lack of the entire nose, nasal airways, olfactory bulbs, and olfactory nerve with the term total arhinia and to use instead the definition partial arhinia or hyporhinia when at least one nostril and olfactory nerve are present [7, 8]. In this definition, proboscis lateralis is not included because this entity is considered as a nasal fusion defect, not an embryological defect of the nasal placodes. Additionally total arhinia could be associated with the absence of the maxillary sinuses and variable penetrance of defects of the palatal, orbital, lacrimal, and central nervous systems [7].

Even if classifying a case into a single category can be complicate and combinations of more defects could be found, according to the most recent comprehensive grouping system of all the congenital nasal anomalities [9], complete or partial arhinia are classified as type 1 which includes atrophy or underdeveloped skin, subcutaneous tissue, muscle, cartilage and bone. These forms are also associated with many craniofacial syndromes such as Opitz G/BBB Syndrome that causes the absence of the nasal cartilage [9].

### 3.2. Relevant Embryology

The nose has a complex development. Comprehension of the mechanism of arhinia and the other nasal anomalies should be based on the study of normal embryology; severe anomalies are normally due to early defect in embryonic life.

Embryological development of the nose occurs between the 3rd and 8th weeks of gestation. During the third and fourth weeks of intrauterine life, two nasal placodes originate from ectodermal thickenings on the frontonasal prominence. In the course of the 5th week, the nasal placodes invaginate into the mesenchyme to form the depressions that will become the nostrils. The mesenchymal cells surrounding the nostrils proliferate, developing the lateral and medial processes, forming the nasal septum. The frontonasal prominence will lead to the development of the nasal dorsum and the glabella. The nose structure is mostly completed by the 8th week [1].

Therefore the embryological defect of the nose is a developmental failure at 4-5th weeks of gestation that impair migration of the nasal placode [10, 11]. It is postulated that the failure of nasal development results from reduced growth of the medial and lateral nasal processes, but it is also possible that overgrowth and premature fusion of the nasal medial process result in the formation of the atretic plate [12]. Arrest of absorption of the nasal epithelial plates at the 13th through the 15th week may be another possible mechanism [7–10]. Additionally, signaling of fibroblast growth factor 8 (FGF8) is one of the important pathways in the process of nasal development [13] and its deficiency in the midfacial area is linked to severe malformations [14, 15].

### 3.3. Etiology and Genetics

Most documented cases of congenital arhinia or hyporhinia appear to be sporadic, isolated and of unknown origin. While research has explored the genetic mechanisms involved in nasal development, specific genes associated with arhinia have not yet been identified [4]. However, certain genes have been suggested as potential candidates, including PAX6, its downstream targets, genes in the FGF signaling pathway, as well as MSX1, NRP2, GSC, ALX3, and ALX4 [16, 17].

Certain studies have noted chromosomal abnormalities involving chromosome 9, although specific loci have yet to be pinpointed [7–18]. Variations observed include mosaic trisomy 9, an inversion on chromosome 9, a reciprocal translocation of *t*(3:12) (*q*13.2; *p*11.2), and variants in the SMCHD1 gene [19, 20].

Similar SMCHD1 variants have been identified in individuals with type 2 Facioscapulohumeral muscular dystrophy (FSHD2) and in those with Bosma arhinia microphthalmia syndrome (BAMS). It remains unclear, however, why FSHD2 and arhinia manifest as separate conditions despite both being linked to pathogenic variants in the SMCHD1 gene. One hypothesis suggests that the DUX4 locus may interact with additional factors, acting as a switch that influences the development of one phenotype over the other [21].

Literature also suggests an association with other craniofacial malformations, primarily due to mutations in the TCOF gene [22], which plays a role in the development of structures that form facial bones and tissues.

Additionally, recent studies have demonstrated that craniofacial abnormalities, including unfused ear pinnae, can be triggered by the ectopic expression of the Hox-1.1 homeobox gene in transgenic mice [23].

Normal karyotype is found in the greater number of the cases, but isolated arhinia may have a familial inheritance too, even if not necessary all members of the family express the malformation in the same way. A case of two sisters with arhinia and microphthalmia born to nonconsanguineous parents is reported and an autosomal recessive mode of inheritance has been suggested in the family [24]. Association with a syndromic pattern, such as Treacher Collins Syndrome or BAMS, has also been reported [25]. BAMS (Bosma Arhinia Microphthalmia Syndrome) is characterized by anomalies of the nose, eye defects (high risk of coloboma and cataract) and delayed puberty (hypogonadotropic hypogonadism) [5]; this syndrome is caused by the presence of SMCHD1 gene variants on chromosome 18.

Also environmental factors have been linked to congenital nose defects, like fetal exposure to oral anticoagulants during pregnancy [26, 27]; an additional case was reported after exposure to escitalopram oxalate, consequently the pregnancy was terminated at the 29th week [10]. Gestational diabetes, polyhydramnios, and fever during pregnancy were also reported [28–30].

### 3.4. Clinical Presentation

The management of a child presenting at birth with arrhinia/iporhinia benefits from the coordinated involvement of a multidisciplinary team of health care professionals. Clinical manifestations of arhinia/iporhinia can infact vary from airway obstruction and inability to feed, to restricted midface development and psychological implications.

In the neonatal period, respiratory and feeding issues constitute the primary concerns [31], due to the fact that newborns are obligate nasal breathers for at least the first 6 weeks up to the first 6 months of life. In case of severe risk of airway obstruction, a pediatric otolaryngology can be present at birth. Airways should be stabilized early, thanks to tracheostomy, oropharyngeal tube or early surgical approach [31–33]. In mild cases, no surgical procedures to maximize airflow are required [34].

Feeding difficulties, secondary to impaired simultaneous sucking and breathing, can cause cyanosis, and may be overcome by the placement of an orogastric tube or a gastrostomy tube [31–33].

Clinical examination of the newborn by the neonatologist involves a careful inspection and palpation of the nose, but also a complete physical examination and instrumental evaluation is recommended to identify any associated dysmorhic feature or malformation. Associated systemic abnormalities may have major implications for the child and for which he/she may need considerable input from general or specialist paediatric services.

Clinics should check for eye (coloboma of the iris, micro or anophthalmia, cataract, nasolacrimal duct atresia, optic nerve atrophy), ear (anotia, microtia), gonads (cryptorchidism, inguinal hernia, micropenis, hypospadia, hypogonadotropic hypogonadism), cardiac (patent ductus arteriosus), or cerebral (holoprosencephaly, meningocele, encephalocele) malformations [6, 31–35]. Cleft or arched palate, choanal atresia, hypertelorism, micrognathia, deficient taste, and smell are frequently associated [31–35]. Unilateral or bilateral small maxilla, with absence of septum, vomer and canine eruption can lead to vertical restriction in maxillary growth [36].

### 3.5. Diagnosis

Congenital arhinia/iporhinia is generally diagnosed after birth, during the first physical examination in the neonatal period, even if the first case of arhinia detected by ultrasound during pregnancy was reported in 2000 [28–31] and since then it was witnessed to further progressing in the field of prenatal diagnosis of arhinia. Prenatal diagnosis is important for genetic counseling, organizing medical assistance after delivery and for possible discussion about pregnancy termination [31]. Two- and three-dimension fetal ultrasound, facial profile, and sometimes fetal MRI (as confirmatory choice in case of inconclusive first-line radiological exams), are used to detect congenital arhinia by 12–16 weeks of gestational age [31, 35–37]. Sometimes fetuses with arhinia accumulate extra amniotic fluid during pregnancy, probably due to swallowing impairment, could lead to premature labour due to excessive stretching of the uterus. Thus, regular ultrasound examination at least every 4 weeks should be recommended. In addition, prenatal genetic screening can detect pathogenic gene variants associated [31].

At birth, in order to establish a specific aetiology, a careful family and medical history is required. Physical examination by the neonatologist and instrumental evaluation are completed by the otolaryngologic assessment and, if possible, in partial arhinia, by the nasal and superior airway fibroscopy. In the postnatal period CT scan or MRI are recommended to detect associated anomalies and midline defects [38]. Furthermore, radiological evaluation could be useful to study nasal, ocular and brain regions to follow face growth and to plan surgical correction. Further investigations like cardiac, renal and cranial ultrasounds, vision and hearing testings, endocrinological evaluation should be performed. Genetic counselling and genetic analysis using Array-CGH could identify copy number variants, while clinical exome could investigate craniofacial syndromes (e.g., Apert, Binder, Treacher Collins, or hemifacial microsomia) that should be always considered too [1].

### 3.6. Theraphy

Mild cases only require nasal hygiene and use of topical steroids, while, considering the limited number of cases described, the surgical timing and approach of severe conditions has not been standardized [1].

Many surgeons prefer performing reconstructive surgery during preschool years or adulthood to achieve further or maximum development of facial structures [39]. However, nasal reconstruction was also performed in newborns or before 1 year of age depending on the severity of the malformation [40, 41].

Generally, a multidisciplinary team with otolaryngologist, plastic surgeons, and prosthodontists is involved [35]. The aesthetic and psychological impact (especially on social integration) of the surgical procedure on children and their families should not be undervalued [42].

Nasal cavities are reconstructed first, either with dental drill and silicone tubes or with maxillary osteotomy (Le Fort II). Then, external nose is created, most frequently using forehead expansion and flap elevation, costochondral grafts and long-term nostril splinting [37]. Use of nasal methyl methacrylate prosthesis from a stereolithographic model is also reported [6]. Vertical distraction osteogenesis, with or without Le Fort III osteotomy, can be performed in order to elongate the mid-face, allowing better face proportion and adequate maxillary height to reconstruct the nasal cavities [35, 36].

### 3.7. Prognosis

Prognosis depends on the malformations and comorbidities associated, but if there are no associated anomalies, then cognitive development is typically normal and patients can lead a regular life [31]. In fact, most cases do not require respiratory support at birth, but orthodontic and/or speech therapy later in life [35]. However, in literature, three cases of infants died in the first 2 months of age are reported too: in one case the baby had respiratory failure 2 hours after delivery, in the other two cases the neonatal course was complicated by sepsis at 29 days and 10 weeks of life, respectively [33]. When necessary, parental training, including eventual tracheostomy care, is recommended [42] because it could improve the prognosis. When iporhinia/arhinia is associated to holoprosencephaly, the prognosis is generally poor.

### 3.8. Conclusions

The nose is a distinguishing feature of the human face. Nasal deformities can cause not only functional problems but also cosmetic and psychological disease. Severity of arhinia/iporhinia depends on clinical manifestations at birth and on existence of associated malformations, in our case the neonate did not present with other anomalies or any specific complication such as respiratory and feeding issues. Even if the neonatal course is uncomplicated, an early multidisciplinary approach is necessary with pediatric otolaryngology and neurosurgeons always involved. We also recommend 3D-CT reconstruction as an effective complementary tool to study the mid face and to confirm the absence of nasal bones to make an accurate diagnosis. Genetic diagnosis and investigations could sometimes greatly assist in providing a diagnosis and informed genetic counselling. The pediatrician is often the primary caregiver for children with arhinia/iporhinia, and as such can coordinate the multidisciplinary inputs needed to offer optimal care for these individuals, including respiratory and feeding issues and family support services.

## Data Availability

All information is available in the clinical records archive of the IRCCS Ospedale Policlinico San Martino.
